# Systemic conditioned medium treatment from interleukin-1 primed mesenchymal stem cells promotes recovery after stroke

**DOI:** 10.1186/s13287-020-1560-y

**Published:** 2020-01-21

**Authors:** Catriona J. Cunningham, Raymond Wong, Jack Barrington, Sabrina Tamburrano, Emmanuel Pinteaux, Stuart M. Allan

**Affiliations:** Lydia Becker Institute of Immunology and Inflammation, Division of Neuroscience and Experimental Psychology, School of Biological Sciences, Faculty of Biology, Medicine and Health, Manchester Academic Health Science Centre, The University of Manchester, AV Hill Building, Manchester, M13 9PT UK

**Keywords:** Cell therapy, Conditioned medium, Mesenchymal stem cell, Secretome, Stroke

## Abstract

**Background:**

Mesenchymal stem cells (MSCs) hold great potential as a therapy for stroke and have previously been shown to promote recovery in preclinical models of cerebral ischaemia. MSCs secrete a wide range of growth factors, chemokines, cytokines and extracellular vesicles—collectively termed the secretome. In this study, we assessed for the first time the efficacy of the IL-1α-primed MSC-derived secretome on brain injury and functional recovery after cerebral ischaemia.

**Methods:**

Stroke was induced in male C57BL/6 mice using the intraluminal filament model of middle cerebral artery occlusion. Conditioned medium from IL-1α-primed MSCs or vehicle was administered at the time of reperfusion or at 24 h post-stroke by subcutaneous injection.

**Results:**

IL-1α-primed MSC-derived conditioned medium treatment at the time of stroke led to a ~ 30% reduction in lesion volume at 48 h and was associated with modest improvements in body mass gain, 28-point neurological score and nest building. Administration of MSC-derived conditioned medium at 24 h post-stroke led to improved nest building and neurological score despite no observed differences in lesion volume at day 2 post-stroke.

**Conclusions:**

Our results show for the first time that the administration of conditioned medium from IL-1α-primed MSCs leads to improvements in behavioural outcomes independently of neuroprotection.

## Background

Stroke is a significant global health problem leading to around 6.7 million deaths annually [1]. For the 33 million people living with stroke, treatment options are very limited and do not fully alleviate the disability caused [2]. There is hence a great demand for regenerative therapies to promote repair and improve disability after ischaemic stroke.

There is a substantial body of evidence showing that mesenchymal stem cells (MSCs) improve functional outcomes in rodent models of cerebral ischaemia, as previously reviewed by Satani and colleagues [3]. In phase I/II clinical trials, MSCs have been suggested to be a safe and feasible therapy for ischaemic stroke [4, 5]. While it was initially thought that cell replacement was the main mechanism of action, it has since been shown that most MSCs become entrapped in the lungs after systemic administration and only a small percentage of those that successfully migrate to the ischaemic brain engraft and differentiate [6, 7]. More recently, interest has shifted towards the paracrine actions of MSCs, also referred to as the bystander effect. The MSC secretome has been implicated in promoting recovery by preventing cell apoptosis, modulating the inflammatory response and enhancing endogenous repair mechanisms such as neurogenesis and angiogenesis [8]. In support of this, the administration of MSC-derived extracellular vesicles [9, 10] and conditioned medium (CM) [11–13] has been shown to improve recovery in rodent models of cerebral ischaemia. Clinical application of such acellular approaches is gaining much interest as potential regenerative therapies as they circumvent the risks of immune rejection and tumorigenesis associated with cell transplantation [14].

A number of in vitro preconditioning strategies have been explored to enhance the MSC secretome including 3D culture [15] and hypoxic preconditioning [16]. In our previous work, we clearly demonstrate that priming with interleukin-1 alpha (IL-1α) drives the MSC secretome towards a more anti-inflammatory and pro-trophic phenotype which may translate into a better therapy for ischaemic stroke [17]. At present, the effect of such MSC preconditioning strategies has not been extensively studied in preclinical models of stroke. Indeed, to the best of our knowledge, there is only one such study, which assessed the efficacy of CM derived from hypoxia-preconditioned MSCs [13] on motor recovery and neuroprotection in a rat model of ischaemic stroke. Our previously published in vitro data clearly demonstrates that IL-1α priming drives MSCs towards a pro-reparative phenotype. Hence, it is essential to confirm the benefit of IL-1α priming in a relevant in vivo paradigm with an extensive characterisation of the post-stroke behavioural repertoire, as investigated here in a mouse model of middle cerebral artery occlusion.

## Methods

### MSC characterisation

For characterisation, MSCs were stained using the BD Stemflow™ hMSC Analysis Kit (BD Biosciences, UK) according to the manufacturer’s instructions. Cells were then analysed on a FACSVerse flow cytometer (BD Biosciences, UK) for positive expression of surface markers CD73, CD90 and CD105 and negative expression of CD11b, CD19, CD34, CD45 and HLA-DR, as defined by the International Society for Cellular Therapy as the minimum criteria for MSCs [18] (Additional file 1: Figure S1). Positive gates were set using fluorescence minus one controls. The multipotency of the MSCs was also assessed using a commercially available kit (R&D Systems, UK). In brief, MSCs were cultured for 21 days in differentiation media with media changes every 2–3 days. Adipocytes were stained with Oil Red O (Millipore, UK), and osteocytes were stained with Alizarin Red (Millipore, UK). Chrondocyte pellets were cut into 30-μm sections using a freezing sledge microtome (Bright Instruments, UK) then stained with toluidine blue (Sigma-Aldrich, UK). Images were acquired using an inverted microscope (Olympus CK X31) and a Moticam 2300 camera connected to Motic Images Plus 2.0 ML software (Motic, Hong Kong).

### Mesenchymal stem cell culture

Passage 5–6 human bone marrow-derived MSCs from a 22-week-old foetal donor (3H Biomedical, Sweden) were used for all experiments. MSCs were cultured as a monolayer in tissue culture flasks (Corning, UK) in MesenPRO RS medium (Invitrogen, UK) supplemented with 1% penicillin/streptomycin and 2 mM glutamine. The growth medium was changed every 4–5 days until the cells were 70–80% confluent. MSCs were then dissociated with 0.5% trypsin-EDTA (Sigma-Aldrich, UK) and counted. For IL-1α-primed CM (αCM) preparation, MSCs were seeded in 6-well plates (Corning, UK) at a density of 1.75 × 10^5^ cells/well and incubated for 24 h. MSCs were then treated with 10 ng/ml human recombinant IL-1α (R&D Systems, UK) for 5 min. Cells were washed twice with PBS then serum-free MesenPRO RS medium (without supplement) was added. After 24 h, αCM was collected, cell debris was removed using 0.22-μM syringe filters (Millipore, UK) and 10× concentrated using 3000 MWCO Vivaspin centrifugal concentrators (Generon, UK) according to the manufacturer’s instructions. For the vehicle, serum-free MesenPRO RS medium was also concentrated 10×. For all in vivo experiments, 400-μl conditioned treatments derived from 3.5 × 10^5^ cells were prepared in advance and stored at − 80 °C.

## In vivo experiments

### Animals

All animal procedures were carried out in accordance with the revised Animals (Scientific Procedures) Act 1986, under a Home Office (UK) project licence, and approved by the local Animal Welfare Ethical Review Board. Animals were group housed in Sealsafe Plus Mouse individually ventilated cages (Techniplast, Italy) at 21 ± 1 °C, 55 ± 10% humidity on a 12-h light-dark cycle. All cages were supplied with Sizzle Nest nesting material (Datesand Ltd., UK) and cardboard enrichment tubes (Datesand Ltd., UK). Mice had ad libitum access to standard rodent diet (SDS, UK) and water. Mice were acclimatised to the facility for at least 1 week before the commencement of experimental work.

### Middle cerebral artery occlusion (MCAO)

**Fig. 1:**
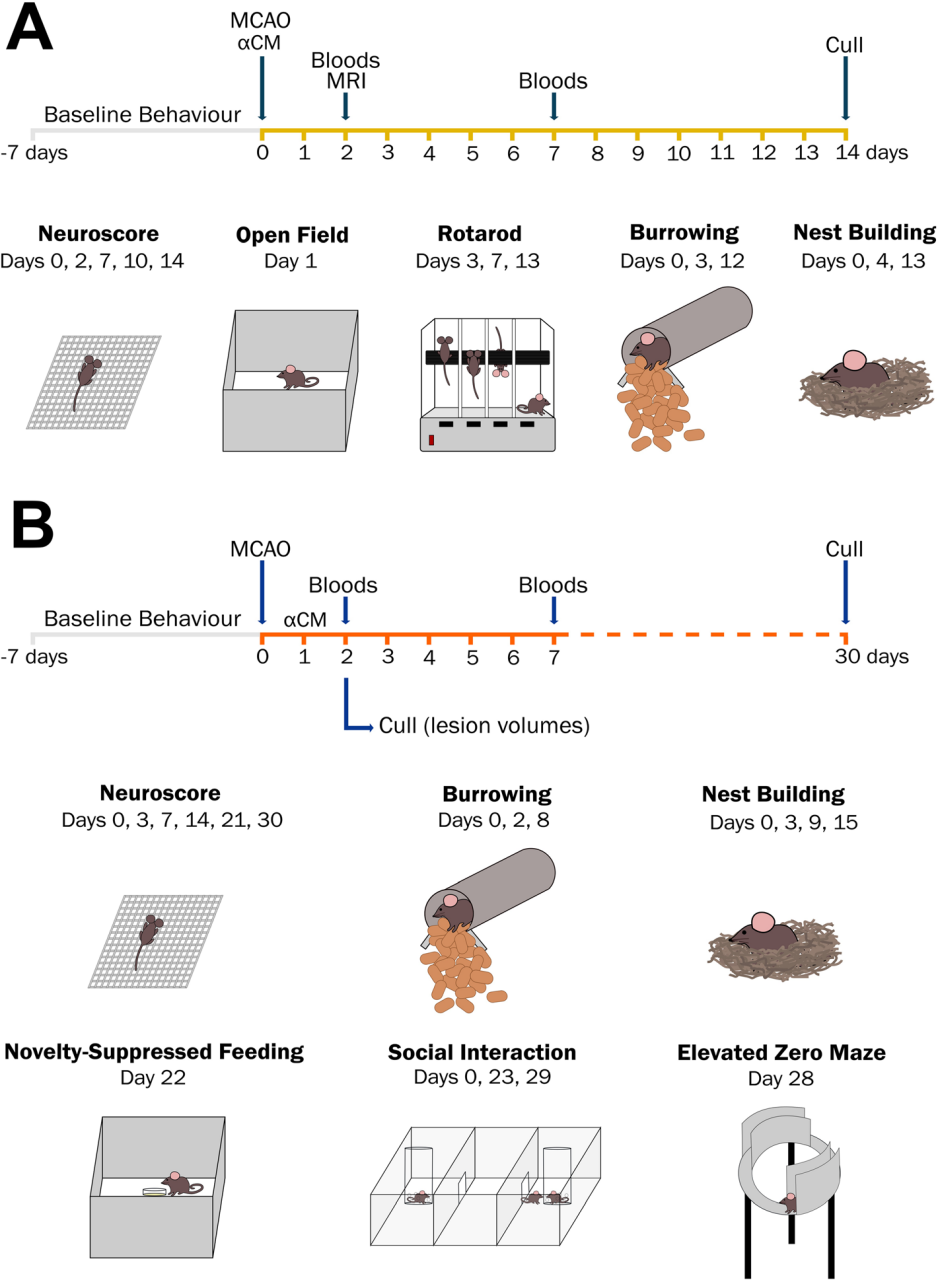
Summary of stroke study timelines and behavioural tests. In the first study, a conditioned medium from IL-1α-primed mesenchymal stem cells (αCM) was administered at the time of reperfusion by subcutaneous injection, then mice were recovered for 14 days (**a**). In the second study, a conditioned medium was administered 24 h post-stroke (**b**). Mice were then recovered for 30 days to facilitate the assessment of post-stroke anxiety and depressive-like behaviours at late time points. With the exception of open field, novelty-suppressed feeding and elevated zero maze baseline behavioural assessments were conducted before stroke surgery

A total of 106 male C57BL/6 mice (Charles River Laboratories, UK) aged 12–20 weeks (mean body weight 27.0 g; 22.5–32.1 g) were used for all stroke experiments. For study 1 (Fig. 1a), the groups were sham + vehicle (*n* = 10), sham + αCM (*n* = 10), stroke + vehicle (*n* = 12) and stroke + αCM (*n* = 12). In study 2 (Fig. 1b), the groups were sham + vehicle (*n* = 12), sham + αCM (*n* = 12), stroke + vehicle (*n* = 12) and stroke + αCM (*n* = 12). For the quantification of lesion volume, additional 14 mice (stroke + vehicle, *n* = 7; stroke + CM, *n* = 7) were culled at 48 h. The groups were randomly assigned using the random number function in Excel. All researchers were blinded to the treatment group during surgery, behavioural assessment and all other analyses. This was achieved by having an independent researcher conceal treatment groups. Transient cerebral ischaemia was induced using the intraluminal filament model of MCAO as previously described [19]. In brief, animals were anaesthetised with isoflurane (4% in 30% O_2_ and 70% N_2_O), and an incision was made in the left common carotid artery. A 6-0 silicon-coated nylon filament (Doccol, USA) was then inserted into the internal carotid artery and advanced to occlude the middle cerebral artery. A laser Doppler probe (Oxford Optronix, UK) was used to confirm a reduction of cerebral blood flow. In study 1, the filament was withdrawn after 20 min to allow reperfusion. In study 2, the occlusion time was reduced to 15 min. In sham animals, the same procedure was performed with the exception that the filament was advanced but withdrawn immediately. Throughout the procedure, body temperature was monitored and maintained at 37 ± 0.5 °C using a homoeothermic blanket and a rectal probe (Harvard Apparatus, UK). Buprenorphine (0.05 mg/kg) was administered via subcutaneous injection at the time of surgery and at 24 h. Mice were weighed daily for the first week post-stroke, given mashed diet and administered with 0.5 ml saline daily until body mass stabilised (usually days 3–4 post-stroke).

A 400-μl 10× concentrated αCM treatment or MesenPRO (vehicle) was administered by subcutaneous injection at the time of reperfusion in study 1 and at 24 h after MCAO in study 2. Animals were excluded from the studies if occlusion was not successful (defined as < 70% reduction in cerebral blood flow) or exceeded humane end points in the IMPROVE guidelines [20]. Details of all exclusions are listed in the “Results” section.

### Behavioural assessment

Tunnel handling was employed as recommended by the National Centre for the Replacement, Refinement & Reduction of Animals in Research (NC3Rs). Prior to the baseline behavioural assessment, mice were habituated to this handling method for 3 days. All behavioural tests were run during the light phase (6 am to 6 pm) unless otherwise stated. Mice underwent a battery of behavioural tests (Fig. 1), conducted by a researcher blinded to the treatment groups. All apparatus were cleaned thoroughly with 70% ethanol between animals to eliminate olfactory cues. Importantly, in contrast to most preclinical stroke studies that focus largely on sensorimotor deficits, here we extend the battery of tests used to assess post-stroke behaviour to include ethologically relevant tests that might correlate with complications such as depression and fatigue, commonly reported by stroke survivors as the most debilitating.

#### Open field

Open field was conducted at 24 h post-stroke in study 1 to assess motor impairment and exploratory behaviour. Mice were placed into the centre of a square Perspex arena (450 × 450 × 200 mm) and allowed to explore freely for 5 min. Distance travelled, time spent in different zones and rotations were analysed live using ANY-maze v4.9 software (Stoelting, USA).

#### 28-point *neurological* score

The 28-point neurological scale (neuroscore) as previously described by Clark and colleagues [21] was performed to assess sensorimotor recovery. In brief, 7 items (body symmetry, gait, climbing, circling behaviour, front limb symmetry, compulsory circling and whisker responses) were scored out of 4 where 0 is normal and 4 is significant impairment.

#### Rotarod

Rotarod has been widely used to evaluate motor recovery after cerebral ischaemia [22]. Mice were placed on an accelerating rotarod (Med Associates Inc., USA) which gradually increased in speed from 4 to 40 rpm over 5 min. Latency to fall or three passively accrued rotations were recorded. A total of three trials were conducted with at least 15 min inter-trial intervals, and the mean was recorded and used for analysis. For baseline measurements, mice were trained for three consecutive days prior to stroke.

#### Burrowing behaviour

Burrowing behaviour was used to assess sickness behaviour after stroke. Burrowing tubes were made from 200 mm lengths of 68 mm diameter PVC downpipe as described previously [23]. Mice were placed in individual cages with a burrowing tube containing 150 g standard rodent diet food pellets (SDS, UK). After 2 h, the food remaining in the tubes was weighed and subtracted from the initial weight (150 g) to calculate the mass burrowed. Two baseline sessions were conducted at least 48 h apart prior to MCAO. Mice which burrowed < 30 g at baseline were excluded from the analysis.

#### Nest building

Nest building, a spontaneous home-cage behaviour, was used as an assessment of well-being. Mice were placed in individual cages containing 20 g of Sizzle Nest nesting material (Datesand Ltd., UK) at least 1 h before the onset of the dark cycle. Mice were left overnight, and nests were scored in the morning after the onset of the light cycle. The scoring system, adapted from Gaskill and colleagues [24], was as follows:

0. No manipulation of nesting material
1. No obvious nest site present (majority of nesting material not contained to one quadrant of the cage)
2. Nest present but flat
3. Nest has raised walls ≤ 30 mm in height
4. Nest walls 31–49 mm in height
5. Nest walls ≥ 50 mm in height

Photographs were taken on a smartphone camera (Xiaomi, China). Each quadrant of the nests was scored by an observer blinded to the group and time point then averaged. Blinding was conducted by asking an independent researcher to rename the file.

#### Elevated zero maze

The elevated zero maze (San Diego Instruments, USA) apparatus consisted of a grey plastic 600 mm diameter annular runway elevated 600 mm above the floor. This is divided into four quadrants: two closed arms with 15-mm walls and two open arms. Mice were introduced into one of the closed arms and allowed to explore for 5 min. ANY-maze software was used again for live tracking.

#### Novelty-suppressed feeding

Novelty-suppressed feeding is widely used to assess anxiety and screen novel anti-depressants [25]. This test measures hyponeophagia, inhibition of feeding in response to a novel environment. The apparatus consisted of a square Perspex arena (450 × 200 × 450 mm) with a 35-mm culture dish (Corning, UK) containing 1 g of sweetened condensed milk (Aldi, Germany) in the centre. Mice were introduced into a corner and allowed to explore for 5 min. A digital USB 2.0 CMOS camera (Stoelting, USA) positioned directly above the apparatus connected to a laptop with ANY-maze version 6.0 (Stoelting, USA) was used for video recording and live tracking. Latency to approach food was timed manually from the videos by an observer blinded to the time point and treatment group. The dish was weighed after testing to calculate the mass of the food eaten.

#### Social interaction and social preference test

The social interaction and social preference test apparatus consisted of a Plexiglas box with three 200 × 400 × 220 mm chambers (Ugo Basile, Italy). The two outer chambers, which could be isolated from the central chamber by insertion of two removable doors, contained custom PET interaction cups (diameter 65 mm, height 195 mm, 10 mm holes to allow sniffing). On the 2-day preceding baseline assessment, each cage was allowed to explore the empty apparatus for 5 min to habituate. On the day of testing, mice were placed in the central chamber for 5 min for further habituation. During this time, a non-littermate control mouse (stranger 1) of the same sex and background was placed into one of the interaction cups. The position was randomised between the trials using a smartphone application (Random Number, Saranomy). The doors were then removed, and the test mouse was allowed to explore for 5 min (session I). For the next session, a second control mouse (stranger 2) was placed in the other chamber, and the test mouse was allowed to explore for a further 5 min (session II). ANY-maze facilitated video recording and live tracking. Interaction time with the stranger mice was scored manually from videos by an observer blinded to the time point and treatment group using an online timer (http://jackrrivers.com/program/). Interaction was defined as a direct contact or stretching of the body within 30–50 mm around the cage.

### Magnetic resonance imaging

At 48 h post-stroke, animals in study 1 were anaesthetised with 4% isoflurane and T2-weighted scans were conducted on Bruker Advance III console (Bruker Biospin Ltd., UK) using a 7-T magnet. A total of 14 serial slices with a thickness of 1 mm were acquired. Lesion volumes were measured using ImageJ and corrected from oedema.

### Quantification of lesion volume

At 48 h, a sub-cohort of animals in study 2 was perfused intracardially with 0.9% saline followed by 4% paraformaldehyde (PFA), in 0.1 M phosphate buffer (PB). The brains were removed and post-fixed in 4% PFA for 24 h then transferred to 30% sucrose before being snap-frozen in isopentane. The sections were cut at a thickness of 30 μm using a freezing sledge microtome and mounted on gelatin-coated slides. The sections were then stained with cresyl violet and coverslipped with DPX mounting medium (Sigma-Aldrich, UK). Lesion volumes were then measured using ImageJ and corrected for oedema.

### Immunohistochemistry

Mice were perfused intracardially as described above. The brains were removed, post-fixed in 4% PFA and cut into 2-mm sections before being processed and embedded in paraffin. The sections were cut at 5 μm using a rotary microtome (Leica, Germany) and mounted on Superfrost Plus slides (Thermo Fisher, UK). After deparaffinisation and rehydration with xylene and ethanol, antigen retrieval was conducted by heating the slides in Tris-EDTA buffer (10 mM Tris base, 1 mM EDTA solution, 0.05% Tween 20, pH 9.0) in a water bath at 95 °C for 30 min. The slides were then loaded in Sequenza racks (Thermo Fisher, UK) was washed with TBST (Tris-buffered saline, 0.1% Tween 20). The slides were incubated in primary antibodies diluted in 1% bovine serum albumin (BSA) TBST for 1 h at room temperature. Primary antibodies used were GFAP (1:2000, ab7260) and iba1 (1:2000, ab178846). The slides were incubated with biotinylated anti-rabbit secondary antibody (1:200, Vector Laboratories, UK) followed by Vectastain ABC and Vector Red substrate (Vector Laboratories, UK). The sections were then counterstained with haematoxylin and mounted with DPX (Merck, UK). Brightfield images were acquired on a 3D-Histech Pannoramic-250 microscope slide-scanner using a × 20/0.30 Plan Achromat objective (Zeiss, Germany). The ipsilateral cortex and striatum were defined as the regions of interest (ROI). Images were acquired using Case Viewer software (3D-Histech, Hungary), then total area of positive staining was quantified using ImageJ by an experimenter blinded to the treatment group.

For immunofluorescence, following antigen retrieval as above slides were incubated in primary Ki67 (1:200, BD550609) and NeuN (1:1000, ab177487) at 4 °C overnight. The slides were then incubated in biotinylated anti-mouse (1:500, Vector Laboratories, UK) and Alexa Fluor™ 647 anti-rabbit (1:500) for 1 h 30. A tyramide super boost kit (B40933) was used to amplify Ki67 signal as per the manufacturer’s protocol. The sections were counterstained using DAPI (1 μg mL^−1^, 10 min, D9542) and mounted using ProLong® Gold Antifade Mountant (Thermo Fisher, UK). Images were collected on a Zeiss Axioimager.D2 upright microscope using a × 20/0.8 Plan Apochromat objective and captured using a Coolsnap HQ2 camera (Photometrics) through Micromanager software v1.4.23. Images were then processed and analysed using ImageJ by an experimenter blinded to the treatment group.

### Data and statistical analysis

All data are expressed as mean ± standard deviation (SD). For the stroke studies, a power calculation of the primary measure of performance in the burrowing behaviour task was conducted with previously acquired data using an online calculator (https://jackauty.com/power-calculator/). Using a mean of 81.2, a SD of 25.9, an alpha of 0.05 and a power of 0.8, an *n* of 8 was calculated to detect a 60% improvement. Accounting for a 30% attrition rate, an *n* of 12 was chosen. Statistical analysis was conducted in RStudio Version 1.1.463 (https://www.rstudio.com) using the car, lme4 and lsmeans packages. Assumptions were assessed graphically, and if necessary, data were transformed. Multilevel modelling was used to analyse body mass, burrowing and rotarod data. If statistical significance was achieved (*p* < 0.05), Holm-Sidak post hoc tests were then conducted. Open field, elevated zero maze, novelty-suppressed feeding, social interaction and immunohistochemical data were analysed by two-way ANOVA followed by Holm-Sidak post hoc tests. All remaining data analysis was conducted using GraphPad Prism 7.01 (GraphPad Software Inc., USA). Nest building and neuroscore data were analysed by Mann-Whitney tests, and lesion volumes were analysed by Student’s *t* test. To determine if mice showed preference for interacting with a stranger mouse over an empty cup and a novel stranger over a familiar stranger in the social interaction test, one-sample *t* tests were used to compare the percentage of interaction time to a theoretical mean of 50%. GraphPad Prism 7.01 was used for presenting all data, and statistical significance was defined as *p* < 0.05.

## Results

### Conditioned medium treatment at time of stroke has a neuroprotective effect and promotes improvements in measures of well-being

In study 1, two mice from the stroke + αCM group were excluded due to subarachnoid haemorrhage and one exceeded the humane end point of > 20% body mass loss and was culled early. In the stroke + vehicle group, one mouse was culled early due to weight loss and two were excluded due to < 70% Doppler drop. The excluded animals were not replaced. There were no postoperative complications in the sham-operated groups.

**Fig. 2:**
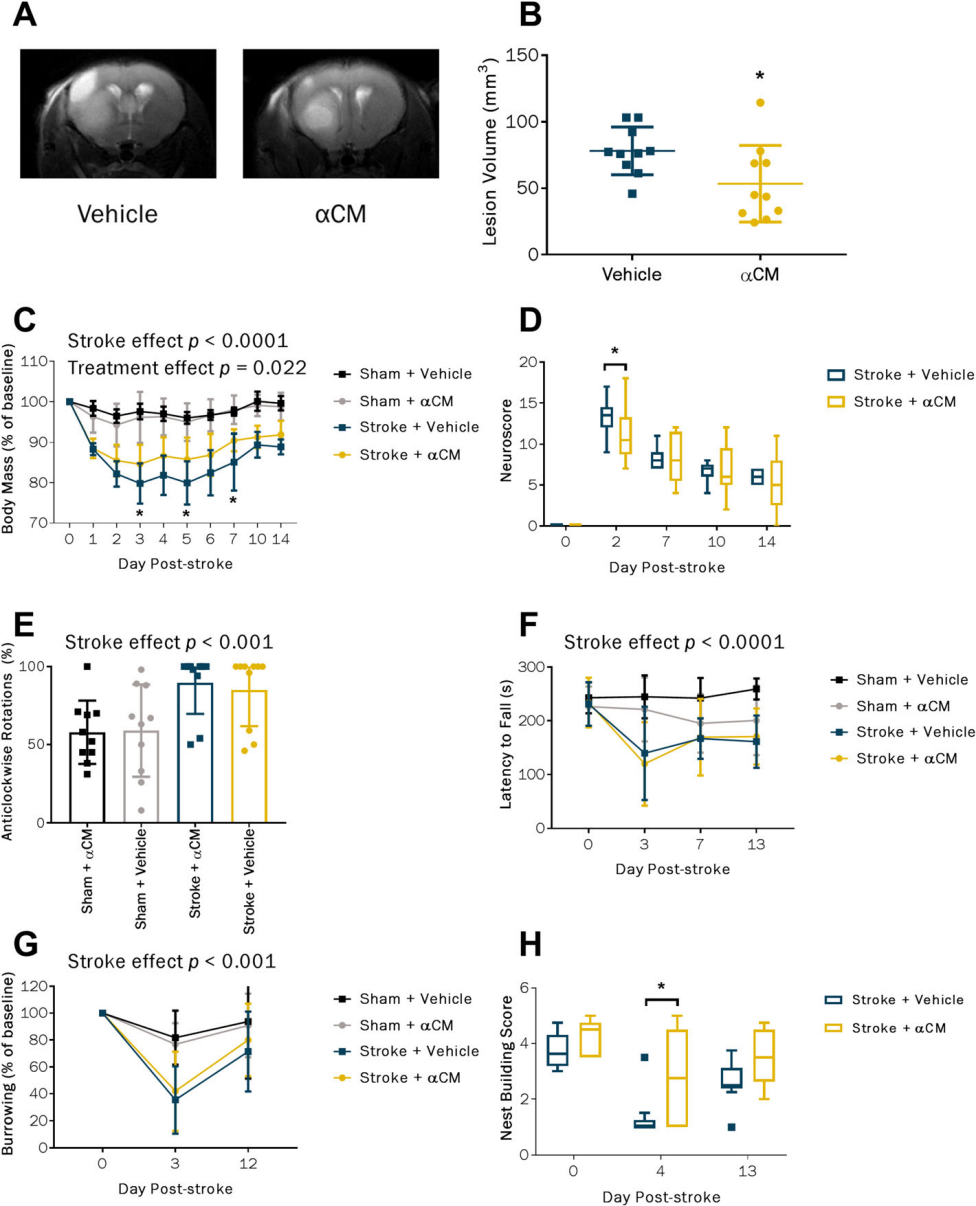
Effect of αCM treatment at the time of stroke on lesion volume and functional recovery. Representative T2-weighted MRI scans (**a**) conducted at day 2 post-stroke and quantification (**b**). Changes in body mass over 14 days post-stroke expressed as percentage of baseline (**c**) and 28-point neurological score (**d**). Percentage of anti-clockwise rotations in the open field test at 24 h post-stroke (**e**) and latency to fall in accelerating rotarod (**f**). Burrowing behaviour (**g**) and nest building (**h**) tests were used as measures of well-being. Neuroscore and nest building data were expressed as median ± IQR. All other data were expressed as mean ± SD. Sham + vehicle, *n* = 10; sham + αCM, *n* = 10; stroke + vehicle, *n* = 9; stroke + αCM, *n* = 9. *p < 0.05

Subcutaneous administration of conditioned medium derived from IL-1α-primed MSCs (αCM) administered at the time of reperfusion had a neuroprotective effect leading to ~ 30% reduction in lesion volume (Fig. 2b). This was associated with modest improvements in well-being. There was an overall treatment effect in body mass loss after MCAO (*p* = 0.022), and post hoc analysis revealed significant differences between αCM- and vehicle-treated mice at days 3, 5 and 7 post-stroke, with αCM-treated animals losing less weight (Fig. 2c). In the 28-point neurological score, the stroke + αCM group performed better than the stroke + vehicle group at day 2 post-MCAO but not at any subsequent time point (Fig. 2d). In the open field task conducted at day 1 post-stroke, there were no significant differences between the treatment groups but there was a stroke effect in percentage of anti-clockwise rotations (*p* < 0.001, Fig. 2e) showing MCAO-induced asymmetry. Furthermore, there were also pronounced stroke effects in accelerating rotarod (*p* < 0.0001, Fig. 2f) and burrowing behaviour (*p* < 0.001, Fig. 2g). At day 3 post-stroke, αCM treatment led to improved performance in the nest building task after MCAO (*p* = 0.04), but by day 13, there was no significant difference compared with the vehicle treatment (*p* = 0.088, Fig. 2h).

At day 14, MCAO was associated with increased expression of microglial marker Iba1 in both the ipsilateral cortex and striatum (*p* < 0.0001, Additional file 2: Figure S2), but there were no differences between the stroke + vehicle and stroke + αCM groups. Similarly, MCAO also led to increased GFAP expression, an astrocyte marker (*p* < 0.0001, Additional file 2: Figure S2), but there was no treatment effect.

### Conditioned medium treatment promotes recovery independently of neuroprotection

From stroke study 2, one mouse from the sham + αCM group was excluded due to post-operative complications. From the stroke + vehicle group, two mice were excluded due to subarachnoid haemorrhage and a further two animals were culled early for reaching humane end points. A total of three mice in the stroke + αCM group were also culled for animal welfare reasons. As in study 1, the excluded mice were not replaced. The overall mortality rate for MCAO in both studies was 19%.

**Fig. 3:**
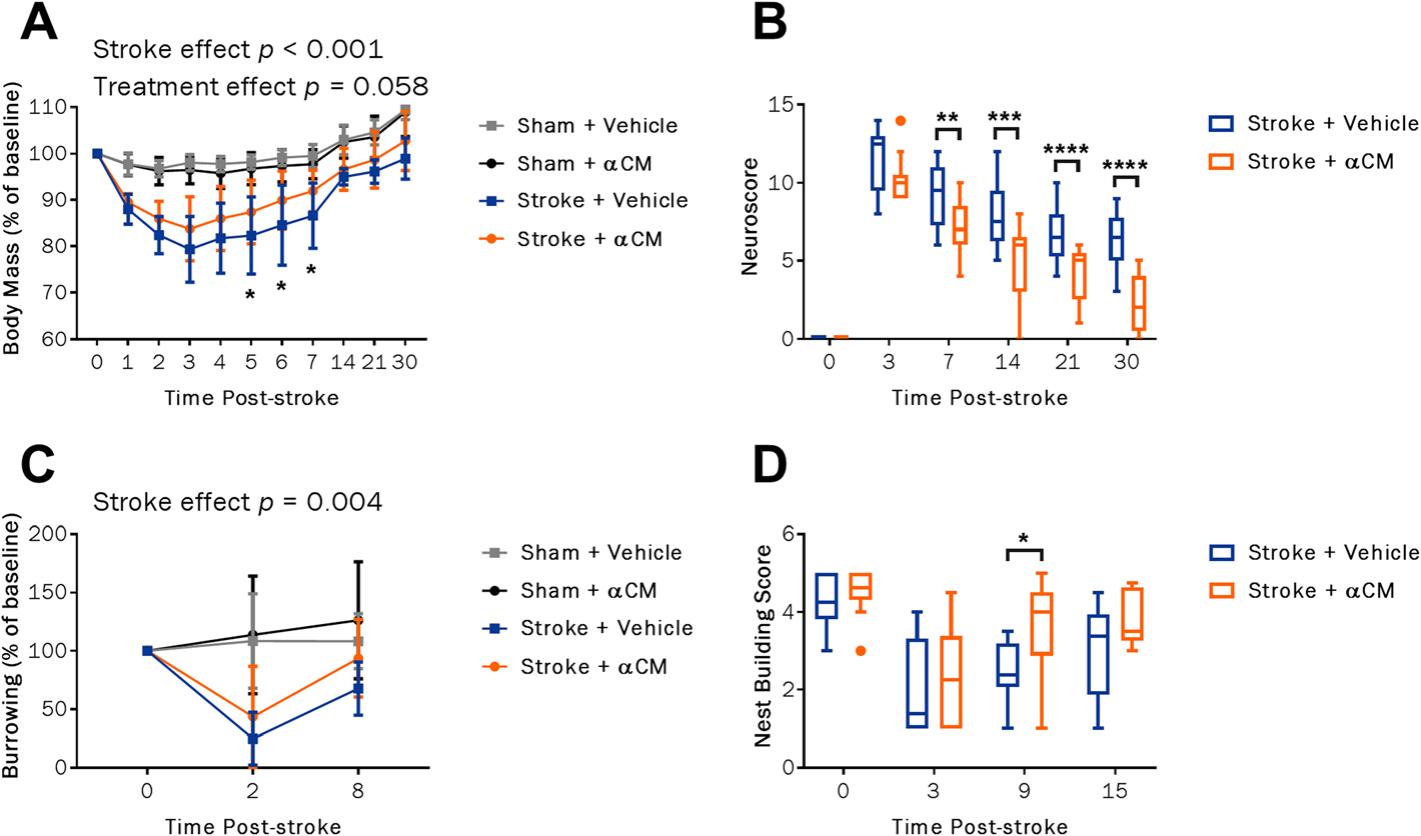
Effect of αCM treatment at 24 h on functional recovery after stroke. Changes in body mass (**a**) and 28-point neurological score (**b**) over 30 days post-stroke. Performance in burrowing behaviour (**c**) and nest building (**d**) tests. Neuroscore and nest building data were expressed as median ± IQR. All other data were expressed as mean ± SD. Sham + vehicle, *n* = 12; sham + αCM, *n* = 11; stroke + vehicle, *n* = 8; stroke + αCM, *n* = 9. *p < 0.05; **p < 0.01; ***p < 0.001; ****p < 0.0001

In this study, the administration of αCM derived from IL-1α-primed MSCs was delayed to 24 h post-stroke. We believe this time point to be outside the window for neuroprotection, and this was confirmed by the lack of effect on lesion volume at 48 h (Additional file 3: Figure S3). While there was no overall treatment effect (*p* = 0.058), there were significant differences in body mass between αCM-treated stroke mice and vehicle-treated animals at days 5, 6 and 7 between the groups, αCM treatment leading to increased weight gain (Fig. 3a). From day 7 post-stroke onwards, αCM treatment was associated with significant improvements in 28-point neurological scores compared with vehicle-treated animals (Fig. 3b). In similarity with the data in the first study, there was no treatment effect in the burrowing behaviour task but there was an acute stroke effect at day 2 (*p* = 0.004, Fig. 3c). At day 9 post-stroke, αCM treatment was associated with improved performance in the nest building task (*p* = 0.0245, Fig. 3d).

At day 30, MCAO was associated with increased microglial Iba1 expression in both the ipsilateral cortex and striatum (*p* < 0.0001, Additional file 4: Figure S4), but there was no treatment effect. MCAO also led to increased GFAP expression in the cortex and striatum (*p* = 0.0002 and *p* < 0.0001, respectively, Additional file 4: Figure S4). Additionally, no differences between the groups were observed in Ki67 staining in the subventricular zone, infarct and dentate gyrus (Additional file 4: Figure S4).

### MCAO induces chronic anxiety-like behaviours

**Fig. 4:**
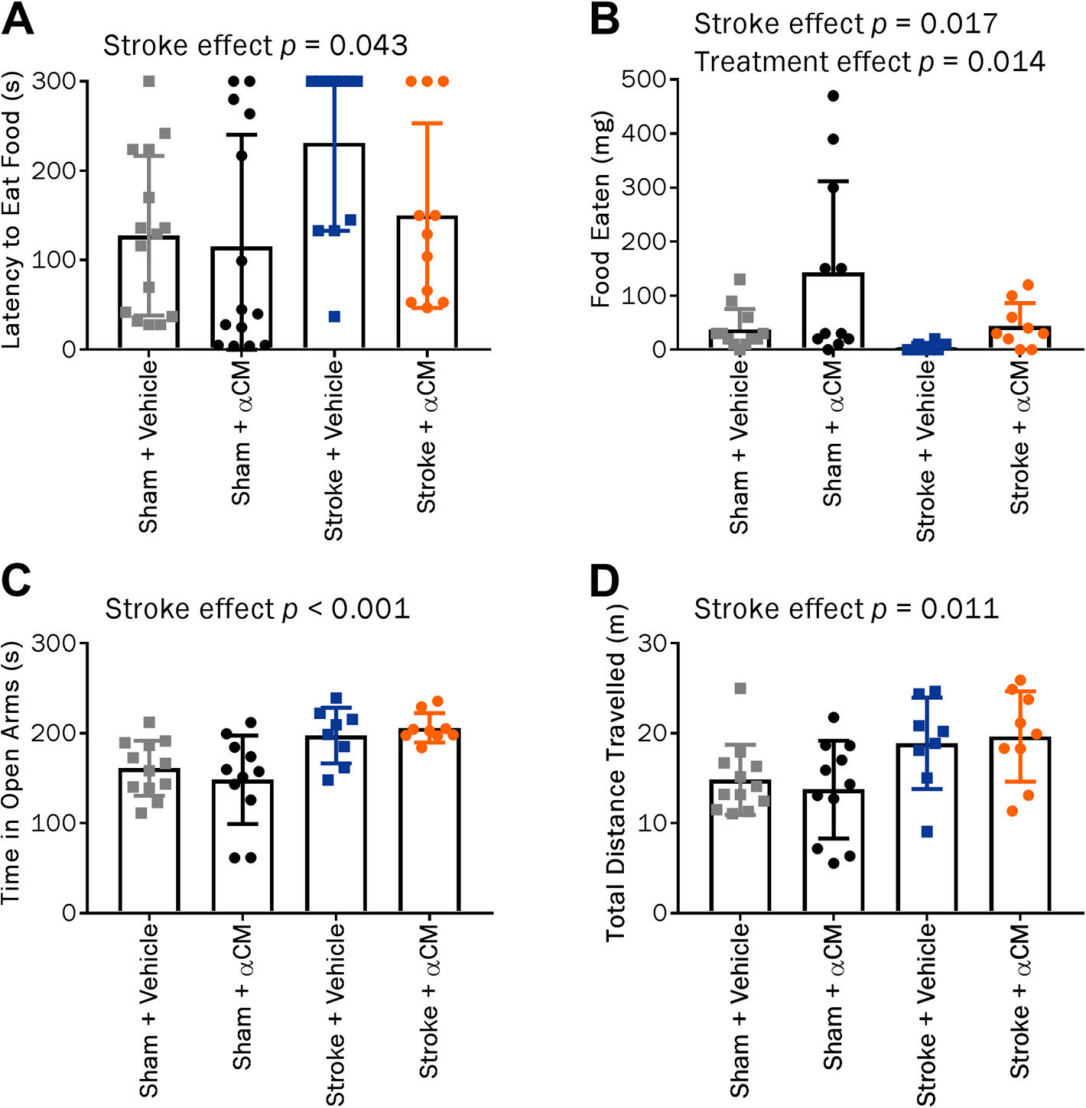
Performance in tests of anxiety and depressive-like behaviours at late time points post-stroke. Latency to eat food (**a**) and mass of food eaten (**b**) in the novelty-suppressed feeding task at day 22 post-stroke. Time spent in open arms (**c**) and total distance travelled in the elevated zero maze at day 28 (**d**). Data were expressed as mean ± SD. Sham + vehicle, *n* = 12; sham + αCM, *n* = 11; stroke + vehicle, *n* = 8; stroke + αCM, *n* = 9

In the novelty-suppressed feeding test conducted at day 22 post-stroke, MCAO was associated with increased latency to eat (*p* = 0.043, Fig. 4a) and decreased food eaten (*p* = 0.017, Fig. 4a) which is indicative of anxiety-like behaviour. Unexpectedly, there was also a treatment effect with αCM being associated with increased consumption of the food (*p* = 0.014, Fig. 4b). In the elevated zero maze test at day 28, stroke spent more time in the open arms of the arena (*p* < 0.001, Fig. 4c) and displayed increased motor activity compared with sham animals (*p* = 0.011, Fig. 4d). The data from the social interaction and social preference test were highly variable, and there were no differences in the interaction time between the stroke + αCM and stroke + vehicle groups at any time point (Additional file 5: Figure S5).

## Discussion

In this study, we provide a detailed in vivo investigation of the efficacy of preconditioning as a strategy to enhance the therapeutic potential of MSCs. More specifically, we demonstrate for the first time that subcutaneous administration of IL-1α-primed MSC-derived CM (αCM) at the time of reperfusion had a significant neuroprotective effect and provided modest improvements in measures of well-being in a mouse model of focal cerebral ischaemia. We then further show that delaying the administration of αCM to 24 h post-stroke led to improved functional recovery, evidenced by increased nest building scores at day 9 and significantly improved neurological scores from day 7 onwards, independently of neuroprotection. Overall, our work indicates that the IL-1-primed MSC secretome could be a useful novel acellular therapy for stroke.

Our results suggest the MSC secretome promotes functional recovery independently of neuroprotection. Since the CM from MSCs rather than the cells themselves is injected, the observed effects must be paracrine. In support of this, a previous study showed that intranasal administration of umbilical cord-derived MSC conditioned medium beginning at 24 h was shown to promote recovery in a rat MCAO model but did not lead to improvements in lesion volume [11]. Delaying the administration of MSCs to 3 days [26], 4 days [27] and 2 weeks post-stroke [28] is also described as effective further suggesting neuroprotection is not the main mechanism of action. As our primary aim was to assess the efficacy of CM, elucidating the mechanisms of action was out of the scope of this study. Indeed, determining the mechanisms from in vivo studies as described here is extremely challenging, as several mediators are involved in promoting recovery which will have different pharmacological effects. For example, neutralising BDNF did not completely abolish the observed improvements in functional deficit following MSC administration in a rat model of ischaemic stroke [28]. Overexpression of glial-derived neurotrophic factor (GDNF), angiopoietin-1 (Ang-1), HGF, FGF1 and PIGF has been shown to further increase recovery compared with non-modified MSCs suggesting these mediators are also involved and highlighting the significant redundancy that exists in such approaches [29–32]. We previously reported G-CSF was a crucial mediator involved in the observed anti-inflammatory effect of αCM in our in vitro cell culture studies, as observed by reduced IL-6 and tumour necrosis factor-alpha (TNF-α) secretion from lipopolysaccharide-stimulated microglia [17]. A logical follow-on study therefore might be to determine the effects of neutralising G-CSF in the αCM on functional recovery after MCAO. However, while this might demonstrate some involvement of G-CSF, it is unlikely to completely abolish the effects of priming, as we saw only a partial effect of G-CSF inhibition in our in vitro studies and also identified the expression of several other potential mediators in the αCM [17]. Though never studied in the context of stroke, TNFα-stimulated gene-6 (TSG-6) has been proposed as a key mediator released from MSCs that can mediate beneficial effects [33], and future studies should consider exploring this further in relation to the actions of the primed MSC-derived CM presented in this study. We see no change in the expression of Iba1 or GFAP with CM treatment which suggests that alterations in glial function are probably not the mechanism involved in CM actions. Similarly, Ki67 expression is unaltered between the vehicle- and CM-treated animals, ruling out any paracrine effects of the CM on neurogenesis, at least under the conditions tested. A multiplex analysis of inflammatory cytokines in the plasma was performed at 2 and 7 days post-stroke to determine whether changes in peripheral inflammation might be responsible for the CM effects, but no consistent differences were observed between the vehicle- and CM-treated groups (data not shown). Despite the lack of mechanism for the CM effect, the overriding primary objective in this study was to provide proof of concept of the therapeutic potential of CM from primed MSCs in stroke, which we believe has been successfully achieved.

In our future work, we would like to explore the mechanisms of action of CM. We acknowledge the chronic end points used in this current work (days 14 and 30) were not optimal for IHC analysis. For example, angiogenesis begins within the first few days with VEGF upregulation in the infarct occurring between 6 and 24 h after MCAO [34]. One potential mechanism to explore is the possible role of cell adhesion molecules (CAMs) in CM-mediated recovery. While not extensively investigated, one study reported that MSC transplantation reduced endothelial ICAM-1 expression which may have been associated with reduced blood-brain barrier disruption [35]. Future studies should therefore investigate the contribution of both vascular- and non-vascular-associated CAMs in the observed effects of IL-1α-primed MSCs. Although we report for the first time that CM from IL-1α-primed MSCs promotes improved outcomes after ischaemic stroke, we do acknowledge that a limitation of this work is the lack of comparison between CM from primed and unprimed MSCs. However, our previously described in vitro cell culture studies clearly demonstrate the added benefit of the IL-1α priming, and our ongoing parallel studies comparing the intra-arterial administration of primed and unprimed MSCs confirm that priming enhances efficacy (Wong et al. unpublished observations).

Mood disorders after stroke are very common and can cause more disability than motor complications. Around 18% of stroke patients experience anxiety [36] and 30–50% are diagnosed with depression [37], both of which are associated with poorer outcomes after rehabilitation. Similarly, fatigue leads to poorer quality of life, and a recent systematic review demonstrated that 50% of stroke patients report fatigue [38]. Despite this evident clinical problem, there is a paucity of literature assessing the efficacy of therapies on non-motor complications in preclinical models of stroke. For this reason, we employed a battery of behavioural tests to assess well-being, depressive and anxiety-like behaviours. To the best of our knowledge, we are the first group to implement such burrowing and nest building tests in a rodent stroke model. Burrowing behaviour has previously been used in mouse models of systemic inflammation, prion disease, cytotoxic hippocampal lesions and Alzheimer’s disease [39–42]. In this study, we demonstrated that stroke induced pronounced deficits in the burrowing task acutely at days 2 and 3 but not at later time points. Burrowing behaviour could therefore be a useful test for assessing post-stroke sickness behaviour. In similarity with burrowing, nest building is a spontaneous home-cage behaviour. Both male and female mice will build nests for thermoregulation [43]. Nest building has previously been used to assess well-being in a number of disease models [44] but not cerebral ischaemia. In this study, we observed disruptions in the nest building behaviour from day 3 to 9 post-stroke in vehicle-treated controls which was partially rescued by αCM. Therefore, nest building appears to be a sensitive test for assessing the efficacy of new treatments on well-being after stroke.

Here, we reported that MCAO induced anxiety-like behaviours from day 22 post-stroke onwards. This is in support of a previous study which showed increased latency to eat in the novelty-suppressed feeding test at 14 weeks post-stroke in a mouse MCAO model and at 19 days following endothelin-induced prefrontal cortex lesion [45]. While we reported increased time spent in in the open arm of the elevated zero maze in contrast to what has been previously reported [45–47], this could be due to hyperactivity. Hyperactivity has been observed as late as 8 weeks after MCAO in mice [48].

## Conclusions

In summary, our results demonstrate for the first time that systemic administration of CM from IL-1α-primed MSCs promotes improvements in recovery in a mouse model of cerebral ischaemia independently of neuroprotection. While there is much research that needs to fully elucidate the mechanisms of action and define which mediators are essential in promoting repair, the IL-1-primed MSC secretome holds much potential as an acellular therapy for the treatment of ischaemic stroke.

## Supplementary information

**Additional file 1: Figure S1.** Characterisation of MSCs. Flow cytometry showing positive staining for MSC surface markers CD90, CD105 and CD73 (A). Cells were negative for CD34, CD11b, CD19, CD45 and HLA-DR (A). MSCs were successfully differentiated down adipogenic lineages as shown by oil red staining for lipid deposition (B). Osteogenic differentiation was evidenced by positive alizarin staining for calcium deposits and toluidine blue staining for cartilage showed differentiation into chondrocytes (B). Scale bars are 25, 100 and 500 μm respectively.

**Additional file 2: Figure S2.** Iba1 and GFAP immunohistochemistry at day 14 post-stroke. There were no treatment effects but stroke led to a significant increase in Iba1 expression (*p*<0.0001) in both the ipsilateral cortex and striatum (A). Representative 20x brightfield images of Iba1 staining (B). Stroke also led to increased GFAP expression (p<0.0001) in the ipsilateral cortex and striatum (C-D). Scale bars = 100 μM. All data expressed as mean ± SD. *n*=4-7. Data analysis was performed by two-way ANOVA and Holm-Sidak post hoc tests.

**Additional file 3: Figure S3.** Lesion volumes at 48 h post-stroke from study 2. Data expressed as mean ± SD. Stroke + vehicle, *n*=7; stroke + αCM, *n*=6.

**Additional file 4: Figure S4.** Immunohistochemistry at day 30 post-stroke. Quantification and representative 20x brightfield images for iba1 (A) and GFAP (B). Ki67 staining in the subventricular zone (SVZ), infarct and dentate gyrus (C). Scale bars = 500 μM. All data expressed as mean ± SD. n=4-12.

**Additional file 5: Figure S5.** Social interaction and social preference at baseline and post-stroke. At baseline, all groups showed preference for interacting with a novel stranger versus an empty cup (A). However, in session 2 the stroke + αCM did not show a preference for the more novel stranger 2. At day 23 post-stroke, there was a treatment effect in session 1 but not session 2 (B). Session 2 at day 29 post-stroke was the only time point at which the stroke + αCM showed preference for the novel stranger (C). Data expressed as percentage of time spent with stranger 1 versus empty cup (session 1) and time spent with more novel stranger 2 versus stranger 1 (session 2). Data expressed as mean ± SD. Sham + vehicle, *n*=12; sham + αCM, *n*=11; stroke + vehicle, *n*=8; stroke + αCM, *n*=9 *, *p*<0.05.

## Data Availability

The datasets used and/or analysed during the current study are available from the corresponding author on reasonable request.

## Abbreviations

CM: Conditioned medium
hMSC: Human mesenchymal stem cell
IL-1α: Interleukin-1 alpha
MCAO: Middle cerebral artery occlusion
MRI: Magnetic resonance imaging
MSC: Mesenchymal stem cell
NC3Rs: National Centre for the Replacement, Refinement & Reduction of Animals in Research
PB: Phosphate buffer
PFA: Paraformaldehyde
SD: Standard deviation
TBST: Tris-buffered saline, 0.1% Tween 20
αCM: Conditioned medium derived from IL-1α-primed cells
