# Supplementary figures and images for: Systemic conditioned medium treatment from interleukin-1 primed mesenchymal stem cells promotes recovery after stroke
Source: Stem Cell Res Ther. 2020 Jan 21;11:32. doi: 10.1186/s13287-020-1560-y (PMC6975095; doi:10.1186/s13287-020-1560-y)

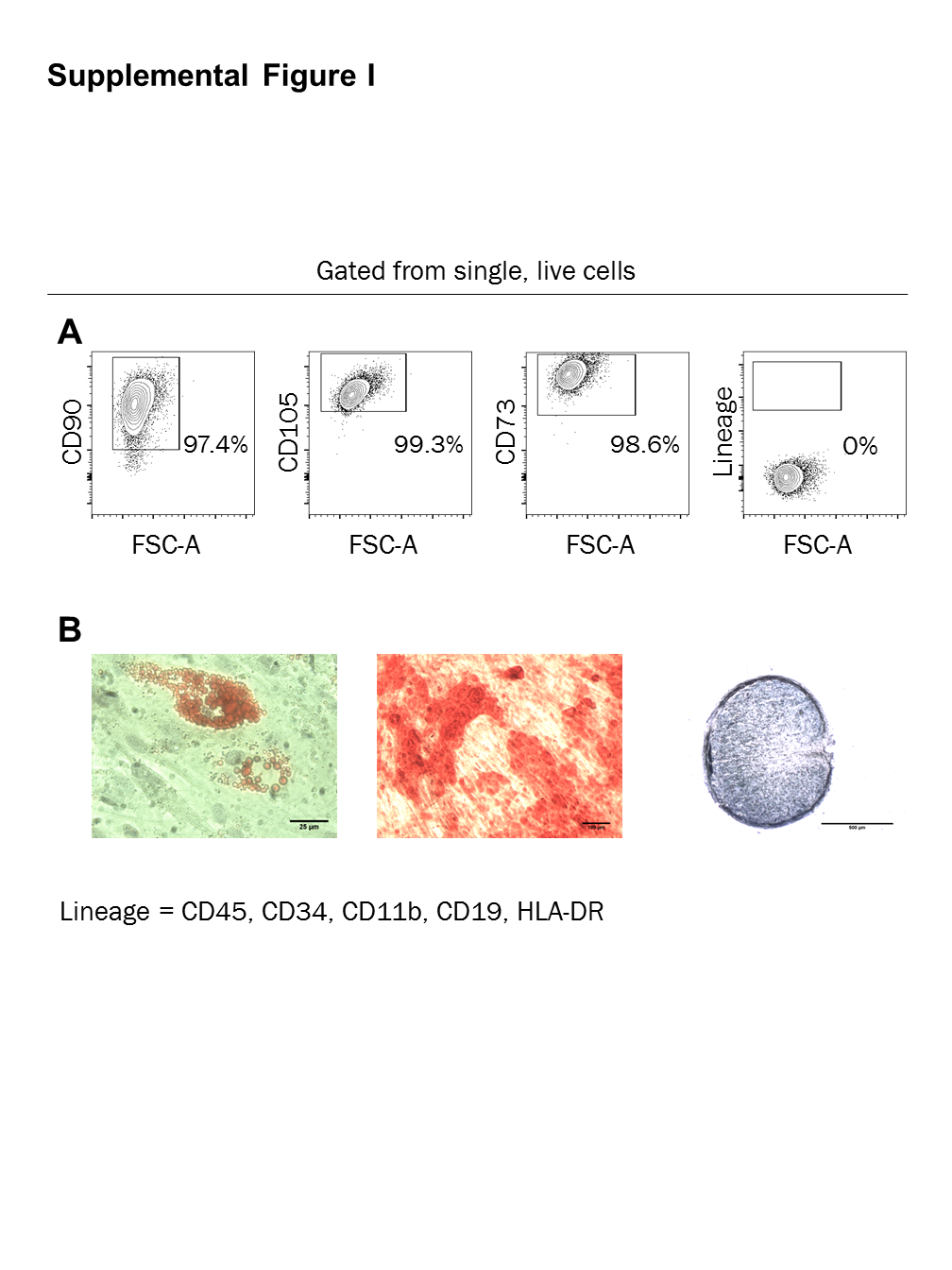

Supplement: Supplementary file 1 — Additional file 1: Figure S1. Characterisation of MSCs. Flow cytometry showing positive staining for MSC surface markers CD90, CD105 and CD73 (A). Cells were negative for CD34, CD11b, CD19, CD45 and HLA-DR (A). MSCs were successfully differentiated down adipogenic lineages as shown by oil red staining for lipid deposition (B). Osteogenic differentiation was evidenced by positive alizarin staining for calcium deposits and toluidine blue staining for cartilage showed differentiation into chondrocytes (B). Scale bars are 25, 100 and 500 μm respectively. [file 13287_2020_1560_MOESM1_ESM.png]

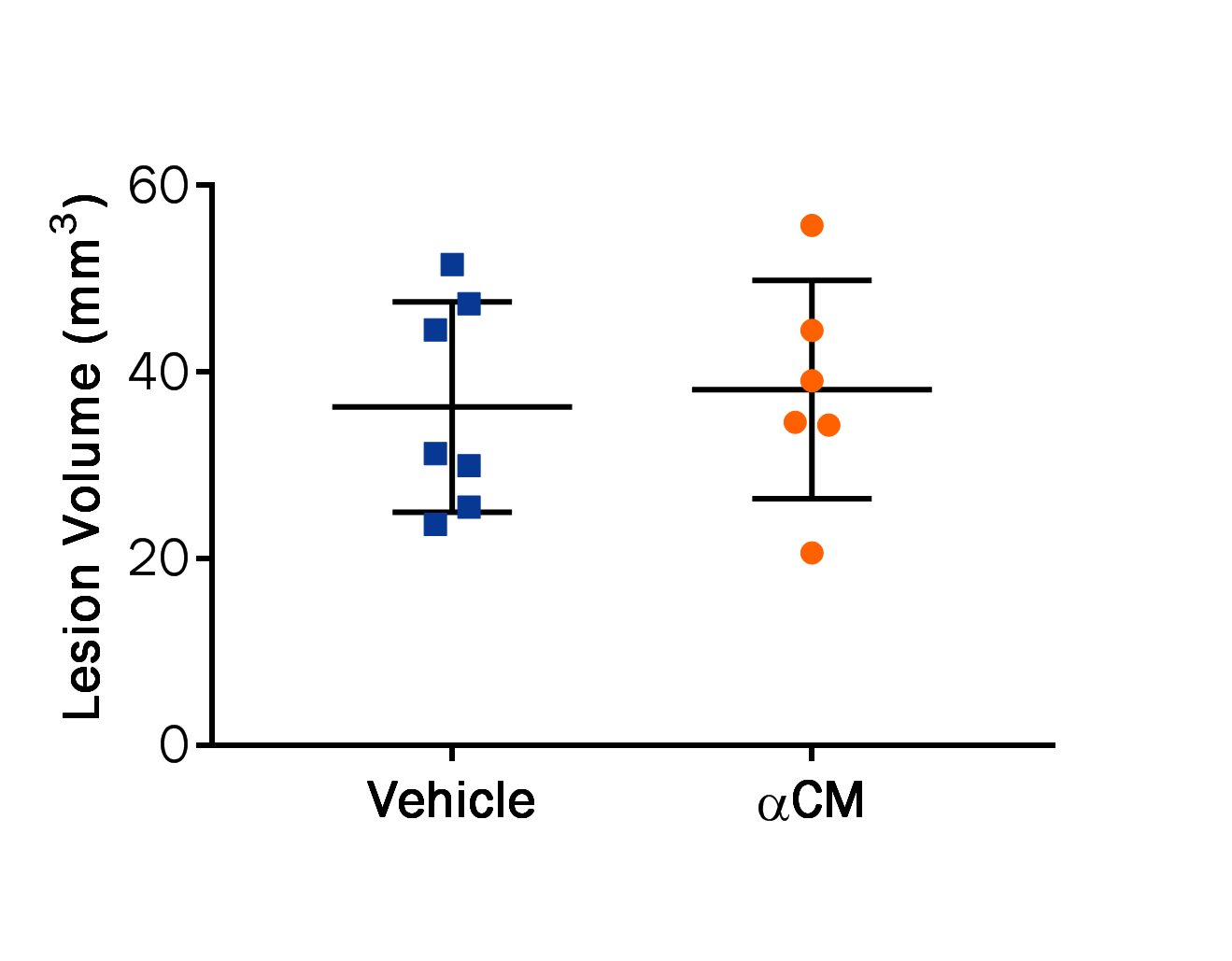

Supplement: Supplementary file 3 — Additional file 3: Figure S3. Lesion volumes at 48 h post-stroke from study 2. Data expressed as mean ± SD. Stroke + vehicle, n=7; stroke + αCM, n=6. [file 13287_2020_1560_MOESM3_ESM.tif]

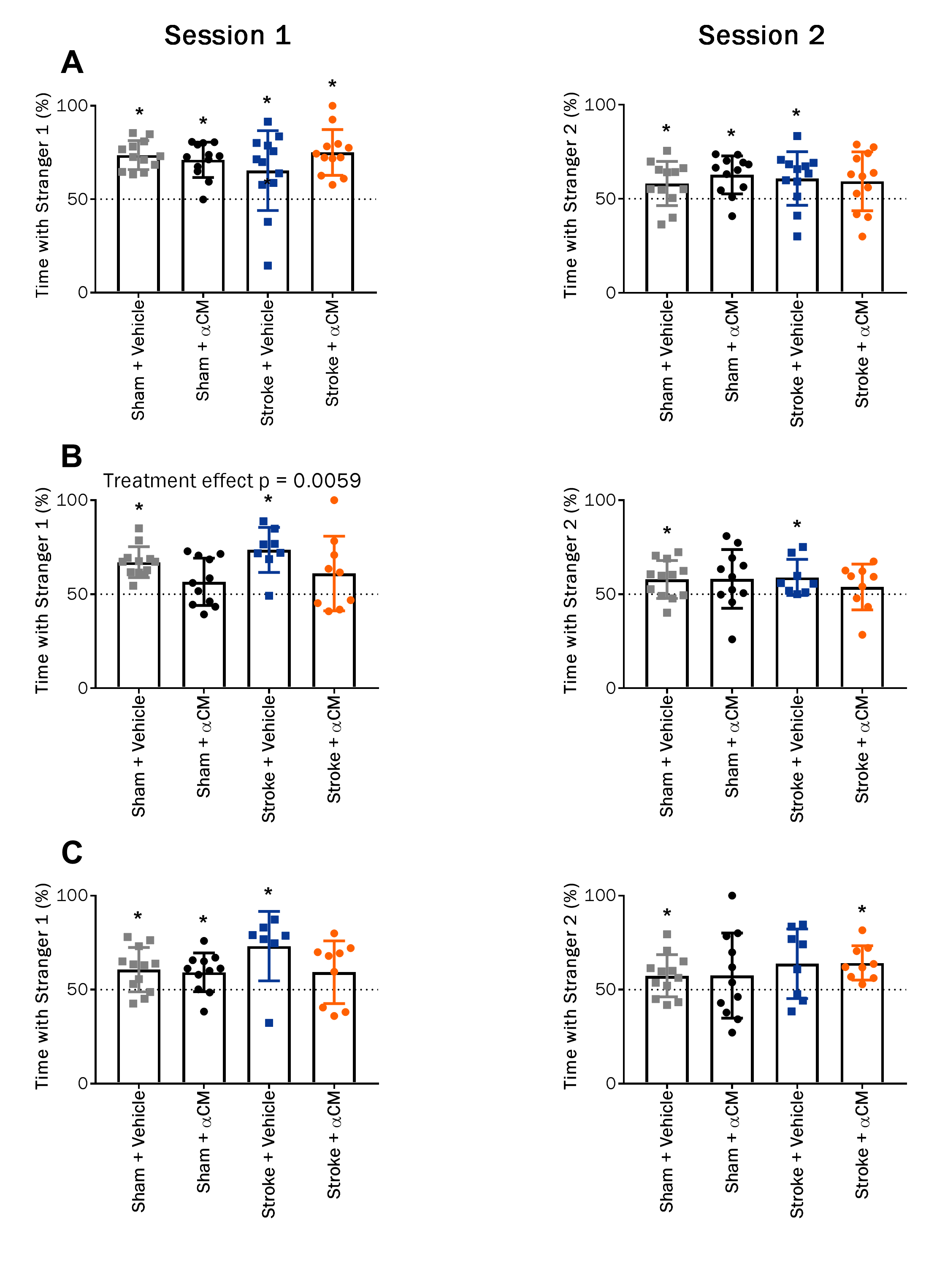

Supplement: Supplementary file 5 — Additional file 5: Figure S5. Social interaction and social preference at baseline and post-stroke. At baseline, all groups showed preference for interacting with a novel stranger versus an empty cup (A). However, in session 2 the stroke + αCM did not show a preference for the more novel stranger 2. At day 23 post-stroke, there was a treatment effect in session 1 but not session 2 (B). Session 2 at day 29 post-stroke was the only time point at which the stroke + αCM showed preference for the novel stranger (C). Data expressed as percentage of time spent with stranger 1 versus empty cup (session 1) and time spent with more novel stranger 2 versus stranger 1 (session 2). Data expressed as mean ± SD. Sham + vehicle, n=12; sham + αCM, n=11; stroke + vehicle, n=8; stroke + αCM, n=9 *, p<0.05. [file 13287_2020_1560_MOESM5_ESM.tif]
